# Basic Psychological Needs Satisfaction Mediates the Link between Strengths Use and Teachers’ Work Engagement

**DOI:** 10.3390/ijerph19042330

**Published:** 2022-02-17

**Authors:** Weitao Jin, Xin Zheng, Li Gao, Zhuolin Cao, Xiaoli Ni

**Affiliations:** 1 Institute of Social Psychology, School of Humanities and Social Sciences, Xi’an Jiaotong University, Xi’an 710049, China; jeason_jin@163.com (W.J.); jolene2022@163.com (Z.C.); 2 Teachers’ Affair Department, Xi’an University of Science and Technology, Xi’an 710699, China; 3 Faculty of Science, Engineering University of the Chinese People’s Armed Police Force, Xi’an 710086, China; xinzheng_781@163.com (X.Z.); gl_li@163.com (L.G.)

**Keywords:** strengths use, work engagement, basic psychological needs satisfaction, teachers

## Abstract

Work engagement is a core indicator that reflects the quality of teachers’ occupational lives and the development of students, but few studies have explored the connection between strengths use and work engagement of teachers and the mechanisms underlying this relationship. This paper aimed to investigate how the relation of strengths use with work engagement is affected by a teacher’s satisfaction of basic psychological needs. For this purpose, 648 teachers in China completed questionnaires. The results revealed that strengths use exhibited a positive correlation with work engagement and needs satisfaction. Furthermore, autonomy, competence, and relatedness satisfaction mediated the effect of strengths use on work engagement for teachers. The results suggest that autonomy, competence, and relatedness satisfaction serve as factors that mediate the effect of strengths use on work engagement. The significance and limitations of the study are discussed.

## 1. Introduction

Teachers are the backbone of college-level education, particularly student development. Promoting teachers’ work engagement and improving the quality of education is the focus of educational theory and practice. Work engagement is a positive and psychologically satisfying mental state marked by vitality, devotion, and immersion [1,2]. Thus, as an important aspect of occupational health [2,3], work engagement has become a key indicator of the quality of teachers’ professional lives and can effectively forecast diverse aspects of teachers’ professional and organizational outcomes, such as task performance, organizational commitment, job satisfaction, active learning and improved teaching to better meet the needs of diverse students [4,5,6]. As a result, in recent years there has been a boom in research concerning the factors that impact teachers’ engagement behavior.

Positive psychology, the promotion of which is on the rise, has given us new insights into strengths use (SU), which refers to the extent to which people apply their own strengths in their daily life [7]. Consequently, research concerning SU has expanded greatly in recent years. Previous research has shown that SU contributes positively to work engagement in different populations [8,9,10,11,12]; yet, little is known about how this relationship pertains to teachers. In addition, the underlying mechanisms that mediate the relationship between SU and work engagement are still largely unknown. To address the shortcomings of previous studies, we evaluated the relationship between SU and work engagement with regard to teachers as well as the potential mechanisms of the connection.

### 1.1. SU and Work Engagement

According to SU theories, it has been demonstrated that employees use the positive psychological states mentioned above as resources to encourage work engagement and proactivity in the work environment [13,14,15]. This suggests that opportunities to apply one’s strengths provide a feeling of professional effectiveness, promote excitement, and heighten motivation. Previous studies have indicated that SU and skill development are strongly linked to work performance as well as personal wellbeing [10,16,17,18]. Considering the observation that only a limited proportion of individuals believe that they identify with and take advantage of their strengths in the professional setting [19], enhancing such strengths is vital to the effectiveness of individual professionals.

Previous studies have indicated that SU correlates positively with the work engagement of civil engineers, website visitors, mining organization employees, Norwegian naval cadets, Dutch employees, and nurses worldwide [10,11,16,18,20,21]. These earlier findings suggest that SU is an important predictor of work engagement. However, little research has been published concerning whether SU affects teachers’ work engagement. As SU theories mentioned, employees can use their strengths in the workplace to encourage work engagement, so we expected that SU would be associated with work engagement in the educational workplace.

### 1.2. The Mediating Role of Needs Satisfaction

In the positive psychology literature, SU has been characterized as a type of positive activity [18]. Studies have indicated that SU correlates positively with work engagement [10,11,16,18,20,21], but few studies have directly explored the underlying mechanism. Therefore, we attempted to uncover the association from the perspective of basic psychological needs satisfaction (BPNS).

BPNS is considered as a fundamental aspect of the self-determination theory, which posits from a humanistic psychological perspective that basic psychological needs consist of autonomy, competence, and relatedness needs [22,23]. The issue of autonomy relates to one’s behavior such as voluntary and wholehearted approval; competence reflects feeling effective, adequate, and creative in individual behavior; and relatedness reflects interpersonal relationships with individuals, particularly loved ones [22,23]. Satisfaction of these three needs is considered to be an important pathway to wellbeing [22,23]. For example, meeting these needs can promote personal growth and livelihood and can significantly affect academic engagement [24,25]. Importantly, previous studies have indicated that BPNS has a positive relationship with work-related wellbeing such as work engagement [26,27,28].

In addition, the positive-activity model states that BPNS is considered as one of four mediating variables linking positive activities such as SU with wellbeing [29]. Consistent with this, empirical studies have found that SU correlates positively with BPNS [30,31,32]. Given that SU, BPNS, and work engagement are closely related, therefore, we hypothesized that BPNS may partly mediate the relationship between SU and work engagement.

### 1.3. The Present Study

We investigated relationships among SU, BPNS, and work engagement in teachers; specifically, we examined the mediator role of BPNS. Our results contribute to the literature in the following ways. First, previous research on SU and work engagement focused primarily on the company’s employees, and little work has focused specifically on teachers. Second, our results contribute to the positive psychology literature primarily by identifying the indirect effects of SU on increasing work engagement as mediated through BPNS—a subject that has not been addressed previously. Finally, we assessed the independent roles of different needs to the association between SU and work engagement using a multiple mediating analysis.

Accordingly, two hypotheses are proposed: (1) SU is substantively related to the work engagement of teachers; (2) BPNS partly mediates the SU-work engagement connection in teachers.

## 2. Methods

### 2.1. Procedure and Participants

The research was conducted at a single university in Xi’an city, central China. The staff of the university includes nearly 1700 teachers. A snowball convenience sampling strategy was used to recruit participants through social media such as WeChat and QQ. The university teachers who were willing to participate in the study could open a link and complete the electronic informed consent form online. The questionnaires to teachers were accessed via a survey website (http://www.sojump.com, accessed on 30 November 2020). A total of 695 teachers completed the questionnaires. Power analysis found that the minimum sample size (*n* = 571) was required to obtain a small correlation (*r* = 0.15, power = 0.95 and α = 0.05) using G*Power 3.1.2. Thus, our sample was adequate. The participants worked and studied at various faculties, including the natural sciences, humanities, and social sciences. Forty-seven teachers who did not answer the questions in a seemingly serious manner were excluded; so, the final sample group comprised 648 respondents for subsequent data analysis. A slight majority of the 648 teachers were female (*n* = 349, 53.86%), and the age breakdown was as follows: 5.71% (*n* = 37) were under 30 years of age, 38.27% (*n* = 248) were 31–40, 37.81% (*n* = 248) were 41–50, 17.90% (*n* = 116) were 51–60, and 0.31% (*n* = 2) were in their 60s.

### 2.2. Measures

The SU Scale was used to assess the level to which each individual utilized their strengths in their professional environment [7]. The SU Scale included 14 items and each item was answered on a 7-point Likert scale from 1 (strongly disagree) to 7 (strongly agree). The Chinese SU is structurally valid [33]. Here, the Cronbach’s alpha was 0.98 for the SU Scale.

To assess BPNS, we used a 9-item measure [34,35] that assessed satisfaction of autonomy, relatedness, and competency. Items were rated on a 7-point Likert scale from 1 (strongly disagree) to 7 (strongly agree). The scale has been shown to be internally consistent in Chinese culture [36]. For this study, the Cronbach’s alpha for the three subscales were 0.68, 0.73, and 0.75, respectively.

Work engagement was analyzed using a 9-item Utrecht Work Engagement Scale [37], which comprises three subscales: vigor, dedication, and absorption. Each subscale comprised three items that were rated on a 7-point Likert scale, from 1 (never) to 7 (always). The revised Chinese version of the Utrecht Work Engagement Scale is structurally valid [5]. For this study, the Cronbach’s alpha for the scale was 0.94.

### 2.3. Data Analysis

Descriptive statistics and Pearson’s correlations were obtained using SPSS 18.0. Mediating analyses were conducted with Mplus 7.0 using the ML estimator and bootstrap method.

### 2.4. Ethics Statement

Informed consent was obtained from each participant. This study was approved by the Institutional Ethics Committee of Xi’an University of Science and Technology (no. 20201130).

## 3. Results

### Correlation between SU, BPNS, and Work Engagement

The kurtosis and skewness of all the items for each scale ranged from −1 to + 1, which indicated the normality of the data [38]. Table 1 lists the Pearson’s correlations among SU, BPNS, and work engagement of the teachers. As expected, SU correlated positively with BPNS (overall), with the three types of BPNS, and with work engagement (*p* < 0.001). In addition, BPNS correlated positively with work engagement (*p* < 0.001).

**Figure 1:**
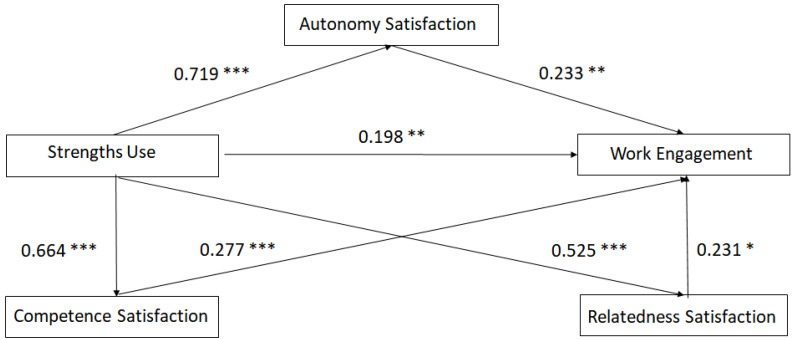
Needs satisfaction mediates the connection between strengths use and work engagement. * *p* < 0.05; ** *p* < 0.01; *** *p* < 0.001.

The results of multiple regression analysis are shown in Table 2. Furthermore, the bootstrap approach (5000 bootstrap samples) was carried out to resolve the statistical significance of the effects of the individual mediators. SU had a significant indirect effect on the teachers’ work engagement via their satisfaction with autonomy, competency, and relatedness (Table 3). This result indicated that satisfaction with autonomy, competency, and relatedness play a mediating function in this link (Figure 1).

## 4. Discussion

The present research aimed to investigate the SU–work engagement association in teachers and the mediating effects of BPNS. The results suggest that SU could affect work engagement via BPNS.

Consistent with findings reported in previous psychology studies, this correlation suggests that SU has a positive relation with work engagement [10,11,16,18,20,21]. This finding is also consistent with SU theories [13,14,15] positing that having the opportunity to use one’s strengths makes one feel effective, energized, and motivated, and these positive mental states as resources help workers to promote both engagement and proactive behavior in the work setting. Furthermore, our results show that increased implementation of SU is associated with increased BPNS, which reinforces previous findings that SU and BPNS are positively associated [26,27,28]. Our results also extend previous research to the educational workplace, suggesting SU plays a crucial role in BPNS in different contexts. Moreover, our findings reinforce those of previous studies showing that BPNS correlates positively with work engagement [29,30,31]. Therefore, our study argues that policy-making must focus on meeting teachers’ BPNS by motivating them to incorporate their strengths professionally. Finally, we found that meeting the three types of BPNS—either separately or in combination—correlated positively with work engagement.

The most novel finding of this study is that SU can consistently predict the probability that teachers will have positive work engagement via the mediating effect of BPNS. This finding accords with the job demands–resources model found by Bakker and Demerouti [39,40], who described that job resources derived from psychological, physical, social, and organizational sources potentiate the ability of workers to achieve their professional goals and promote their performance on the job; i.e., if teachers use their character strengths, they will be more engaged in the educational workplace. Importantly, our results extend the reach of the model by demonstrating that autonomy, relatedness, and competence satisfaction can be mediators of SU and work engagement for teachers. The positive aspect of using one’s own strengths may enhance teachers’ awareness, thereby helping them to: (1) choose a course of action that is consistent with their goals (satisfies autonomy); (2) develop meaningful relationships with students, colleagues, or family members (satisfies relatedness); and (3) achieve a competitive advantage in the educational workplace (satisfies competency); these aspects lead to more substantive work engagement [22,23]. In addition, our findings are in accordance with self-determination theory [22], i.e., that satisfying all three needs is critical for psychological wellbeing. The tenet of broad needs satisfaction can promote mental health and positive behaviors and significantly impact academic engagement [24,25].

## 5. Limitations of the Study

Some potential limitations should be mentioned. First, the research used a cross-sectional design; therefore, we could not track the dynamics of work engagement developed over time with our teacher cohort. Nor did we consider the effects of temporal changes in SU and of BPNS on work engagement. Therefore, an additional, longitudinal design study must explore the dynamics of work-engagement development. Second, the data were collected via a questionnaire. Although the scales we used have been shown to be both reliable and valid, our findings may have been affected by unpredictable social interactions. Therefore, any additional studies should attempt to eliminate or reduce such a potential bias, e.g., via the use of a multiple appraisal method. Third, our sample included only teachers in China, and therefore future studies should incorporate participants from other cultures (including other countries), so that the results acquired from several studies can be generalized across populations.

## 6. Conclusions

Our study is the first to explore the means by which psychological needs satisfaction potentiates the power of SU to enhance work engagement among teachers. It was found that SU correlated positively with teachers’ work engagement. Moreover, satisfaction with each of three key aspects, namely autonomy, relatedness, and competence, could partly explain the link between SU and work engagement with respect to teachers, consistent with the positive activity model. These results have crucial implications for teachers and university administrators. First, considering the direct effect of SU on work engagement, administrators should understand that encouraging teachers to use their strengths increases their work engagement. Therefore, strengths-based counseling or coaching [41,42] may assist teachers use their strengths and remain engaged in the educational workplace. Moreover, administrators should understand that strengths-based counseling/coaching may not be confined to promoting SU; rather, it may also apply to a wider range of education-related outcomes. Second, university managers should foster a positive environment within each academic department that is favorable to addressing the basic psychological needs of teachers. Intervention techniques that are founded on self-determination theory—particularly interventions for autonomy, relatedness, and competency support—can be developed to improve teacher engagement.

## Figures and Tables

**Table 1:** Correlations among the variables.

Variable	M(SD)	Correlation					
		1	2	3	4	5	6
1. Strengths use	63.63(16.47)	1					
2. Autonomy satisfaction	12.79(3.64))	0.567 ***	1				
3. Competence satisfaction	14.55(3.22)	0.410 ***	0.398 ***	1			
4. Relatedness satisfaction	13.91(3.06)	0.488 ***	0.608 ***	0.470 ***	1		
5. Basic needs satisfaction	41.25(8.07)	0.605 ***	0.840 ***	0.757 ***	0.841 ***	1	
6. Work engagement	39.85(9.84)	0.622 ***	0.530 ***	0.470 ***	0.498 ***	0.622 ***	1

Note: *** *p* < 0.001.

**Table 2:** Multiple regression analysis.

Predictors	Outcomes	B	*t*	R2	F
Model 1					
Strengths use	Work engagement	0.372	20.188 **	0.386	407.552 **
Model 2					
Strengths use	Autonomy satisfaction	0.125	17.487 **	0.320	305.784 **
	Competence satisfaction	0.080	11.436 **	0.167	130.784 **
	Relatedness satisfaction	0.091	14.220 **	0.237	202.217 **
Model 3					
Strengths use	Work engagement	0.237	11.006 **	0.475	147.431 **
Autonomy satisfaction		0.424	4..012 **		
Competence satisfaction		0.576	5.663 **		
Relatedness satisfaction		0.385	3.133 **		

Note: ** *p* < 0.01.

**Table 3:** Test of direct and indirect effects.

Model Pathways	Estimated	95%CI		Odd (%)
		Lower	Upper	
Direct effect				
SU → work engagement	0.198	0.036	0.336	29.77
Indirect effect	0.467	0.336	0.613	70.23
SU → autonomy satisfaction → work engagement	0.167	0.050	0.284	25.11
SU → competence satisfaction → work engagement	0.146	0.094	0.212	21.95
SU → relatedness satisfaction → work engagement	0.154	0.046	0.307	23.16
Total effect	0.665	0.598	0.725	1.00

Note: SU = strengths use.

## Data Availability

The datasets analyzed during the current study are available from the corresponding author upon reasonable request.
